# Headache as a Prognostic Factor for COVID-19. Time to Re-evaluate

**DOI:** 10.1007/s42399-020-00657-7

**Published:** 2020-11-26

**Authors:** Paolo Martelletti, Enrico Bentivegna, Michelangelo Luciani, Valerio Spuntarelli

**Affiliations:** 1 Department of Clinical and Molecular Medicine, Sapienza University, Rome, Italy; 2 Emergency Medicine & COVID-19 Unit, Sant’Andrea University Hospital, Rome, Italy

**Keywords:** COVID-19, Headache, Prognostic factors, Drug–drug interactions (DDIs), Emergency medicine, Cytokine release syndrome (CRS)

## Abstract

Headache occurs in only about 13% of patients within the cohort of presenting COVID-19 symptoms. The hypothesis that such a painful symptomatic picture could be considered a prognostic factor for COVID-19 positive evolution or its trend of severity, or the co-generation of hyposmia/anosmia and/or hypogeusia/ageusia, needs robust epidemiological data, punctual pathophysiological demonstrations, and a detailed comparative analysis on drug–drug interactions (DDIs).

## Viewpoint

To date, scientific literature is regrettably and exceedingly generous towards the term “COVID-19”: 71,664 scientific articles published [1], 49,578,590 confirmed cases, and 1,245,717 confirmed deaths for COVID-19 in less than 1 year [2].

As the number of new registered cases continues to rise on a daily basis, 441,696 on the 9th of November 2020, so too is there an increase in questions for the scientific community while certainties struggle to emanate from this immensity of scientific data. It is clear that all systems have been crushed by this cytokine release syndrome (CRS) and that the list of epidemiological and clinical characteristics, including headache, does not appear to be subject to further updates. Headache as a symptom of COVID-19 is present in only 12.9% of patients afferent to COVID-19 emergency medicine [3].

Headache, for those experiencing the COVID-19 clinic at first hand, does not seem to be of such clinical relevance, but only an epiphenomenon secondary to the involvement of complex systems, the cardiovascular/coagulative and respiratory/neurological, other than to a febrile state.

The assumption that the international scientific community should use headache as prognostic factor of COVID-19 duration or severity in a COVID-19 emergency medicine clinical setting is a recent concept. Such assumption is expressed in two recent articles, Caronna and Magdy [4, 5]. The first study has been carried out on 130 COVID-19 patients (74.6% with headache), while the latter on 172 COVID-19 patients with headache selected from a cohort total of 238 COVID-19 patients (52.9%) is the highest percentile reported in the majority of the scientific literature [1]. Interestingly, these data came from the use of face-to-face interviews in patients strongly compromised and in a clinical setting strictly sealed and in operational emergency.

The methodology of the two reports is not the dominant concept now, but we wish to highlight how every subtended COVID-19 disease is scotomized, like radiological and blood coagulation data, parameters on pulmonary, renal, cerebral functionality [6, 7] without stating clear correlations, apart from generic considerations on pathophysiological hypothesis with the clinical manifestations of headache [4, 5].

We must not overlook the fact that in order to curb this unbridled pathology, we are struggling with therapeutic approaches centering on this CRS, applying a variety of drugs with uncertain impact on the multifarious COVID-19 disease and with little known action on headache symptomatology as protease inhibitors, heparin, macrolide antibiotics, hydroxychloroquine, and monoclonal antibodies for CAR T cells [8]. It is not yet known to what extent they could interfere, increase, suppress, or cause alone or through a drug–drug interaction (DDI) any clinical symptom related to headache [8]. We should also be very cautious when conjugating headache with hyposmia/anosmia and/or hypogeusia/ageusia present in COVID-19 patients [9] following a generic concept of proximity [10].

A rigorous big data analysis would help obtain more transparent numbers that headache experts might then translate into verified, steady, and consolidated prognostic indications useful for a clearly different medical scientific community operating in and studying COVID-19 emergency medicine.

## Data Availability

Not applicable.
